# Research on the multi-granularity method of role engineering

**DOI:** 10.3389/fncom.2022.1020277

**Published:** 2022-11-09

**Authors:** Yongmei Jiao, Menghan Zhang, Yu Wu

**Affiliations:** ^1^Sifang College, Shijiazhuang Tiedao University, Shaoxing, China; ^2^The School of Information Engineering, North China University of Water Resources and Electric Power, Zhengzhou, China; ^3^School of Earth System Science, Institute of Surface-Earth System Science, Tianjin University, Tianjin, China; ^4^Tianjin Key Laboratory of Earth Critical Zone Science and Sustainable Development in Bohai Rim, Tianjin University, Tianjin, China

**Keywords:** access control, role-based access control (RBAC), granularity calculation, concept lattice, role system

## Abstract

The role-based access control model (RBAC) is an access control approach oriented to enterprise security policy, which plays a leading role in the field of access control due to its good applicability and flexibility. However, as the scale of access subjects and access objects expands, it becomes more difficult to develop a role engineering system that meets security requirements. Particle size calculation can reduce or improve the particle size of the problem and improve efficiency on the premise of obtaining satisfactory solutions. In this study, the granularity calculation method was introduced into the role formulation process of RBAC, which can effectively reduce the complexity of the problem and improve the efficiency of the RBAC role engineer. At the same time, the concept lattice reduction method was used to reduce the role concept lattice and reduce the workload of the system security administrator.

## Introduction

The development of the information system has made information sharing between people more convenient and fast. However, the explosive growth of the information system has not only provided people with convenient and fast access to information but also created the problem of information security. For example, the “ZTE Incident” caused a huge loss to ZTE because the legal staff had access to confidential documents and information. The disclosure of prism makes people pay more attention to the protection of personal privacy. Ransomware attacks at hospitals, universities, and enterprises and on personal computers uses encryption algorithms to illegally encrypt the files of the victim host, forcing victims to pay a certain ransom to decrypt the files, causing huge losses to enterprises and individuals. An increasing number of security attacks and information leaks have seriously affected cyberspace security, personal privacy, and national security (Michel and King, 2019; Qiu et al., 2020).

To prevent the intrusion of illegal users, access control limits the access ability and scope of the access subject to the access object using predefined methods or policies and limits the access to resources in the information system. Access control consists of an access subject, an access object, and the operation (Fang et al., 2017). In this case, the subject is the user or the access behavior initiated by the user (such as process and thread). The object is the entity that is accessed by the subject, and all the information, resources, and objects that can be regarded as the object in access control, such as files and disks, can be operated. An operation is an action that a subject can perform, such as a read operation and a write operation.

Traditional access control models include the autonomous access control model and the mandatory access control model. In the autonomous access control model, users can freely transfer their permissions, which makes the access control very flexible, but the arbitrary transfer of permissions leads to the risk of illegal access. In the mandatory access control model, the direction of information flow is from a high-security level to a low-security level, which can guarantee that the information system cannot be accessed by illegal elements. However, the mandatory access control model can not be flexibly applied to the current flexible and changeable market environment.

The role-based access control model (RBAC) is considered an access control strategy that can effectively guarantee the security of an information system, which ensures not only the security of the system but also its flexibility. The RBAC model introduces the concept of roles between users and access by assigning (revoking) roles to users. Thus, the purpose of granting (revoking) access permissions is achieved. In this way, system management only needs to consider the granting or revoking of roles, instead of considering the granting or revoking of each permission one by one, which simplifies the work tasks of system administrators. The role discovery method based on concept lattice can obtain the non-redundant role system semiautomatically, but with the increase in the number of access objects in the system, the number of access permissions in the system will increase sharply. In this case, the role-based access control model can reduce the tasks of system administrators to some extent, but the expansion of the number of roles makes the tasks of administrators very complex.

At present, we are seeking a way to ensure the security of the system without being affected by the massive size of the role system, which becomes difficult to manage effectively. Granular computing can be used to study the same question in different granularity of discussion (Qian and Liang, 2006). In the case of ensuring a satisfactory solution, appropriately reducing the role partition granularity can effectively reduce the number of roles and reduce the complexity of the work of the system security administrator.

This study begins with a discussion on the role exploration method based on concept lattice, introduces the concept of granularity calculation into this method, finds out the appropriate calculation granularity, uses the larger granularity, reduces the size of the role system, and thus reduces the complexity of the problem.

## Basic definition

The concept lattice definitions used in this study are as follows (Zhao et al., 2009; Ganter and Wille, 2012; Wei et al., 2020):

**Definition 1:** A context *K* = (*U, M, I*) is composed of two sets *U* and *M* and there exists a relation I between *U* and *M*. The elements of *U* are called objects and the elements of *M* are called attributes. (*u, m*) ∈ *I* or (*uIm*) means that the object *u* has an attribute m. We use (*u, m*)∉*I* to denote that object *U* does not possess property *M*.

**Definition 2:** Let *K* = (*U, M, I*) be a context. For *A* ⊆ *U*, *B* ⊆ *M*, denote *f*(*A*) = {*m* ∈ *M*|∀*u* ∈ *A*, (*u, m*) ∈ *I*} and *g*(*B*) = {*u* ∈ *U*|∀*m* ∈ *B*, (*u, m*) ∈ *I* }. If *A* and *B* satisfy that *f*(*A*) = *B* and *g*(*B*) = *A*, then we call the binary group (*A, B*) as a *concept*, where *A* is the *extent* of the concept (*A, B*) and *B* is the *intent* of the concept (*A, B*).

The set of all formal concepts is denoted as *L* = (*U, M, I*), ∀*u* ∈ *U*, with object concepts as (*g*(*f*(*u*)),*f*(*u*)). Similarly, for ∀*m* ∈ *M*, (*g*(*m*), *f*(*g*(*m*))) is called an attribute concept. In addition, for ∀(*X*_1_, *B*_1_), (*X*_2_, *B*_2_) ∈ *L* = (*U, M, I*), a partial order relationship ≤ can be defined in the sense that (*X*_1_, *B*_1_) ≤ (*X*_2_, *B*_2_) is equivalent to *X*_1_ ⊆ *X*_2_ and *B*_2_ ⊆ *B*_1_.

**Definition 3:** Let (*U, M, I*) be a formal context and if *Y* ⊆ *M* satisfies.

(1) *Y*≠*f*(*g*(*Y*)) (*Y* ⊆ *f*(*g*(*Y*))) and
(2) Each pseudo-intent *Y*_1_⊂*Y* has *f*(*g*(*Y*_1_)) ⊆ *Y*, then, *Y* is called a pseudo-intent.

**Definition 4:** Assume that set *K* = (*U, M, I*) is a context and *Y*_1_, *Y*_2_ ⊆ *M*. If *g*(*Y*_1_) ⊆ *g*(*Y*_2_), then *Y*_1_ → *Y*_2_ is true in *K*.

**Definition 5:** If *K* = (*U, M, I*) is a formal context, then the value dependency set {*X* → *f*(*g*(*X*))|*X* is the pseudo-intent of *K*} is the Duquenne–Guigues of *K*.

**Definition 6:** Given access security context *K* = (*U, M, I*), implication set *J*(*K*), and implication formula *C* → *D* ∈ *J*(*K*), if the attribute set *T* ⊆ *M* is equivalent to *C*⊈*T* or *D* ⊆ *T*, then *T* is related to *C* → *D*. If *T* is related to all the implication forms in *J*(*K*), then *T* is related to *J*(*K*).

**Definition 7:** Let *R*_1_and *R*_2_ be non-empty equivalence relations in the field of argument. If *R*_1_ ⊆ *R*_2_, it is said that *R*_1_ is thinner than *R*_2_ and it is denoted as *R*_2_ ≤ *R*_1_ or *R*_2_≥*R*_1_.

The concept of granular computing involves the theory of granulation, projection, and synthesis operation of quotient space (Wang et al., 2018). Quotient space theory is a kind of theory that can imitate human beings to analyze problems from coarse to fine, from surface to interior, and from multilevel to multidimensional, to effectively reduce the complexity of problems (Li et al., 2017).

The information theory definitions used in this study are as follows:

**Definition 8:** (Fuzzy equivalence relation) Let *R* ∈ *T*(*x*,*x*), if

(1) ∀*x* ∈ *X, R*(*x, x*) = 1;
(2) ∀*x, y* ∈ *X, R*(*x, y*) = *R*(*y, x*); and
(3) $\forall x,y,z,R\left(x,z\right)\geq \;\underset{y}{\sup}\left(\min\left(R\left(x,y\right),R\left(y,z\right)\right)\right)$,

then, R is called a fuzzy equivalence relation.

**Definition 9 (Savchenko**, **2019****):** An information system is a binary *S* = (*U, C*), where *U* = {*x*_1_, *x*_2_, ···, *x*_*n*_} is a set of non-empty finite subject set, called the field of theory, and *C* = {*a*_1_, *a*_2_, ···, *a*_*m*_} is a non-empty finite object set. For any *a* ∈ *C*, satisfying *a*:*U* → *V*_*a*_, i.e., *a*(*x*) ∈ *V*_*a*_, *x* ∈ *U*, then *V*_*a*_ = {*a*(*x*)|*x* ∈ *U*} is called the value range of *a*.

**Definition 10 (Sheng et al.**, **2020****):** For an information system *S* = (*U, C*) and an arbitrary subset of non-empty properties*B* ⊆ *C*, an equivalence relation *R*_*B*_ can be derived on *U*:

*R*_*B*_ = {(*x, y*) ∈ *U*×*U*:*a*(*x*) = *a*(*y*), ∀*a* ∈ *B*}.

Furthermore, a quotient set $\frac{U}{R_{B}}\;can\;be\;formed\;between\;R_{B}$ and *U*, i.e., $\frac{U}{R_{B}}=\left\{{\left[x\right]}_{B}:x\in U\right\}$, where [*x*]_*B*_ = {*y* ∈ *U*:(*x, y*) ∈ *R*_*B*_} is the equivalent class of *x* on attribute *B*.

## Research on the multi-granularity method of role engineering

The role discovery method based on concept lattice can obtain the role system in the access control system in a semiautomatic way, but not all the roles acquired are demonstrative. For example, in a school information management system, there are two roles, namely, teacher information management and teacher information management and scientific research management. These two roles overlap in practice. That is, the system only needs to have both teacher information management and scientific research management roles to meet the requirements of system security. Although the role exploration method based on concept lattice can obtain a complete role system, it does not consider the realistic significance between roles, resulting in the existence of many unnecessary roles.

The solution to the above problem is to further optimize the role system based on concept lattice so that the optimized role system can not only meet the security requirements but also achieve the minimum number, reducing the workload of the system administrator to manage the role. In contrast to the traditional optimization method (Lv and Zhang, 2017; Lv et al., 2018, 2020, 2021a,c; Zhang et al., 2020a), granularity computation, rough set, and multi-granularity decision theory can not only obtain a satisfactory solution to the problem but also effectively reduce the complexity of the problem. Therefore, the idea of granularity can be introduced into the role exploration method based on concept lattice to simplify the role system and reduce the complexity of the work of the system administrator.

### Role optimization method of decision information system

**Definition 11:** The decision system is binary, *s* = (*U*∪{*d*}, *C*), and {*U, C*} is an information system (Qian et al., 2018). Here, *C* is called conditional property, *d*∉*U* is called decision property, mapping *d*:*C* → *V*_*d*_, where *V*_*d*_ is the range of decision property *d* (Yan et al., 2020).

The object *x*_*i*_ takes a unique observation value on the attribute *a*_*j*_, which is the single-granularity information system (Mi et al., 2003). If the object takes different observation values according to different scales in terms of attributes, a multi-granularity information system can be obtained (Du and Hu, 2014). Assuming that the structure of granularity level number is I, from the fine-grained to coarse-grained layered structure, the properties of each layer to $C^{k}=\left\{a_{1}^{k},a_{2}^{k},\cdot \cdot \cdot ,a_{m}^{k}\right\},\left(1\leq k\leq I\right)$, where there is an information system *S*^*k*^ = (*U, C*^*k*^). $a_{j}^{k}\in C^{k}$,where $V_{j}^{k}$is subjective and the range of the attribute *a*_*j*_ layer *k* (Li and Li, 2015). There exists $a_{j}^{k+1}\left(x\right)=g_{j}^{k,k+1}\left(a_{j}^{k}\left(x\right)\right),x\in U$ (Dai and Xu, 2012).

According to the basic concept of the decision system, the role system can be observed from different aspects. Based on this idea, this study proposes the following role optimization solutions.

(1) Based on the view of the multi-granularity hierarchical model, each access role is divided according to the access authority. For the role system, we classified the roles that have obvious inclusion relations, such as (teacher information management) and (teacher information management and scientific research management). The two roles are classified into one category, and so on. The division results are recorded, and the set of division results for each layer is obtained.
(2) On each level, the role exploration method based on concept lattice is incorporated into the conceptual system obtained and is divided according to the access subject. For example, the access subject is (teacher) and (teacher, director of scientific research department). The two types of subjects can be divided into one type and analogically. The division results are recorded, and the set of division results for each level is obtained.
(3) By integrating the division results of steps (1) and (2), the coordination of the decision system is assessed to obtain a multilevel granularity decision model and select the ideal role granularity.

**Figure 1:**
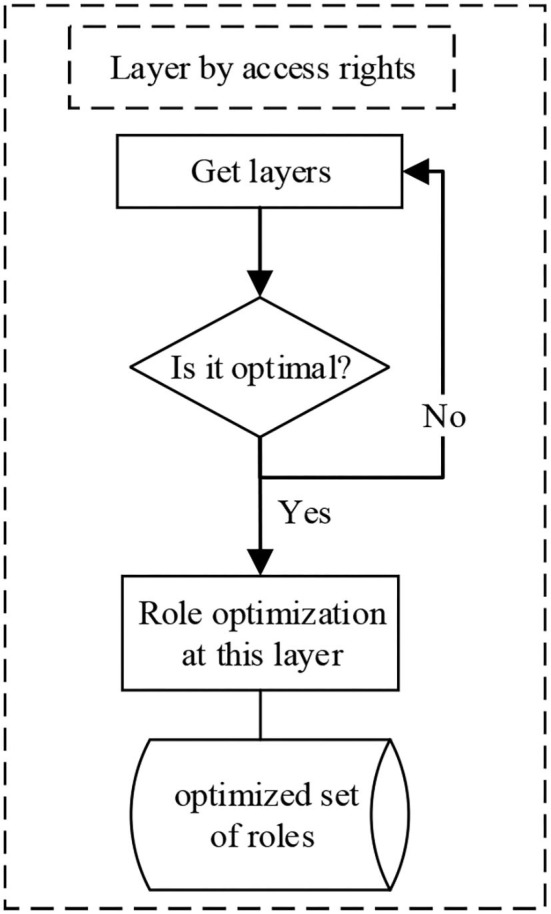
Stratification by access permissions.

The hierarchical division by access is shown in Figure 1.

**Figure 2:**
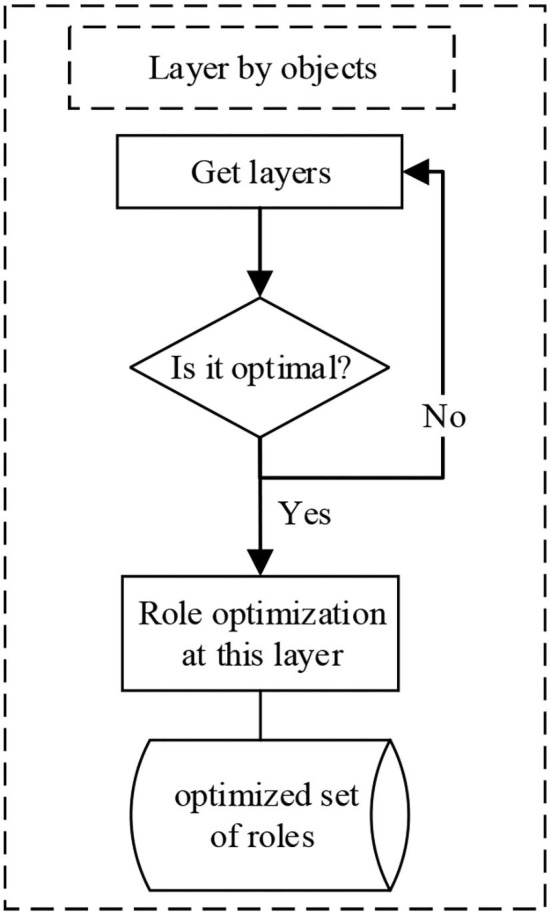
Stratification by access object.

Similarly, the process of hierarchical division according to access objects is shown in Figure 2.

According to the concept of a decision system, the results of each layer division can be verified to obtain an optimal granularity level and then to reduce the number of roles in the system according to the hierarchical relationship.

## Role optimization method for conceptual lattice computing

The “Role optimization method of decision information system” section describes role optimization using the knowledge of decision information theory. This section guides on how to further optimize the role concept lattice from the perspective of the role concept lattice.

According to the basic knowledge of concept lattice, a concept lattice is composed of many concepts in the formal context and then displayed in the form of a lattice.

Example 1: Let *K* = (*U, M, I*) be a formal context, as shown in Table 1.

**Table 1:** Formal context *K*.

	**a**	**b**	**c**	**d**	**e**	**f**	**g**	**h**	**i**
1	1	0	1	1	1	1	1	0	0
2	0	0	0	1	0	1	0	1	1
3	0	0	1	0	0	0	1	0	0
4	0	1	0	1	0	1	0	1	0

**Figure 3:**
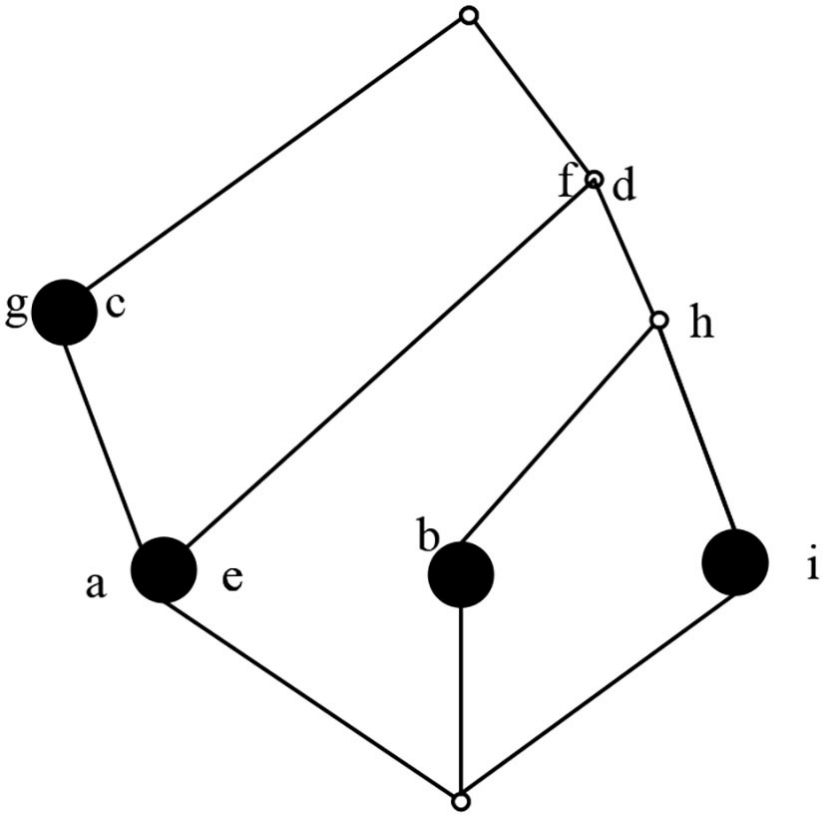
Concept lattice.

According to the calculation method of concepts, the concepts in the computable formal context *K* are (1234, ∅), (124, *df*) (24, *dfh*), (2, *dfhi*), (13, *cg*), (4, *bdfh*), (1, *acdefg*), and (∅, *abcdefghi*). The concept lattice composed of concepts is shown in Figure 3.

As shown in Figure 3, in the concept lattice, all concepts are displayed in a partial order structure. The characteristics of this partial order structure can be used to find concepts that can be optimized. According to the role exploration method based on concept lattice, at the end of exploration, the obtained concept lattice is constructed by roles and users in the system. If the final role concept lattice can be optimized, the role system in the system can be optimized. Based on this idea, combined with the following definition theorem, the role concept lattice can be reduced to a certain extent.

The relevant definitions and theorems to be used are as follows (Zhao, 2018).

**Definition 12:** Let *K* = (*U, M, I*) be a formal context. If for ∀*x*_1_, *x*_2_, ···, *x*_*n*_ ⊆ *U*−*u*, the relationship *g*(*u*)≠*g*(*x*_1_)∩*g*(*x*_2_)…*g*(*x*_*n*_) holds, then *u* is called a granular irreducible object.

**Definition 13:** Let *K* = (*U, M, I*) be a formal context. If for ∀*y*_1_, *y*_2_, ···, *y*_*n*_ ⊆ *M*−*m*, *f*(*m*)≠*g*(*y*_1_)∩*g*(*y*_2_)…*g*(*y*_*n*_) holds, then *u* is called the particle irreducible property.

Based on the above definitions, we have the following conclusion:

**Theorem 1:** Let *K* = (*U, M, I*) be a formal context.

(1) *For* ∀*x* ∈ *U*-*u*, if *g*(*x*)⊃*g*(*u*) does not exist, then *u* is a granular irreducible object.
(2) *For* ∀*y* ∈ *M*-*m*, if *g*(*y*)⊃*g*(*m*) does not exist, then *m* is a granular irreducible property.

Algorithm 1 is a description of the role exploration algorithm based on the concept lattice. The algorithm borrows the attribute exploration algorithm framework in the concept lattice, takes the user as the object in the formal background of the attribute exploration algorithm, and uses the authority as the attribute to construct the formal background. Furthermore, the implication relationship between permissions is used as a problem to interact with the domain experts so as to actively assist the domain experts to construct the RBAC role system and then to construct the role concept lattice. The core process of the algorithm is as follows: First, the implication relationship between permissions is used as a problem to interact with domain experts; second, experts judge whether these questions are valid. Then, according to the different answers of experts, the implication relationship between the next permissions that need to be exchanged is calculated. Finally, the loop generates interaction problems until the algorithm reaches an end condition. At the end of the algorithm, the method can obtain the concept lattice of the role and the implication relation set among permissions of the given information system. However, when the number of users and permissions in the information system is excessive, the role concept lattice is still very large. According to the reducible attribute and reducible object theory, we proposed the *RSA* algorithm (Algorithm 2).

**Algorithm 1:** Role exploration algorithm based on concept lattice description.


**Input:** Attribute set *M*, the decision in the expert's mind about the validity of attribute dependency (interaction in operation).
**Output:** Principal basis *J*_*i*_(*K*), intent set *C*_*i*_(*K*). BEGIN
1: *J*_0_(*K*) ← ∅;*C*_0_(*K*) ← ∅;*K*_0_ ← ∅; *U*_0_ ← ∅;*B*_0_ ← ∅;
2: FLAG = FALSE
3: WHILE(*B*_*i*_≠*M*)
4: *f*(*g*(*B*_*i*_)) is computed in *K*_*i*_
5: *B*_*i*_ → *f*(*g*(*B*_*i*_))−*B*_*i*_ Is this valid in *K*
6: IF no, give a counterexample *u*_*i*_
7: *U*_*i*+1_←*U*_*i*_∪*u*_*i*_
8: *B*_*i*+ 1_ ← *B*_*i*_
9: *J*_*i*+ 1_(*K*) ← *J*_*i*_(*K*)
10: *C*_*i*+ 1_(*K*) ← *C*_*i*_(*K*)
11: *K*_*i*+1_ ← *K*(*U*_*i*+ 1_, *M, I*)
12: ELSE
13: *K*_*i*+ 1_ ← *K*_*i*_
14: IF(*f*(*g*(*B*_*i*_ ))≠*B*_*i*_)
15: FLAG=TRUE
16: *C*_*i*+ 1_(*K*) ← *C*_*i*_(*K*)
17: *J*_*i*+1_(*K*) ← *J*_*i*_(*K*)∪*(B*_*i*_ → *f*(*g*(*B*_*i*_))−*B*_*i*_*)*
18: ELSE
19: *J*_*i*+ 1_(*K*) ← *J*_*i*_(*K*)
20: *C*_*i*+1_(*K*) ← *C*_*i*_(*K*) ∪(*B*_*i*_)
21: END IF
22: *B*_*i*+1_ ← next attribute
23: END IF
24: END WHILE
25: END

**Algorithm 2:** *RSA* algorithm description.


**Input:** Role concept lattice *L*(*U, M, I*)
**Output:** Reduced role concept lattice *L*_1_(*U, M, I*) BEGIN
1: *C*(*K*)= The set of intent in *L*(*U, M, I*)
2: *U*(*K*) = *g*(*C*(*K* ))
3: WHILE(*C*(*K* ))
4: *m*=Reducible attribute
5: *C*(*K*)= *C*(*K* )-*m*
6: ENDWHILE
7: WHILE(*U*(*K* ))
8: *u*= Reducible object
9: *U*(*K*)= *U*(*K*)- *u*
10: ENDWHILE
11: END

**Figure 4:**
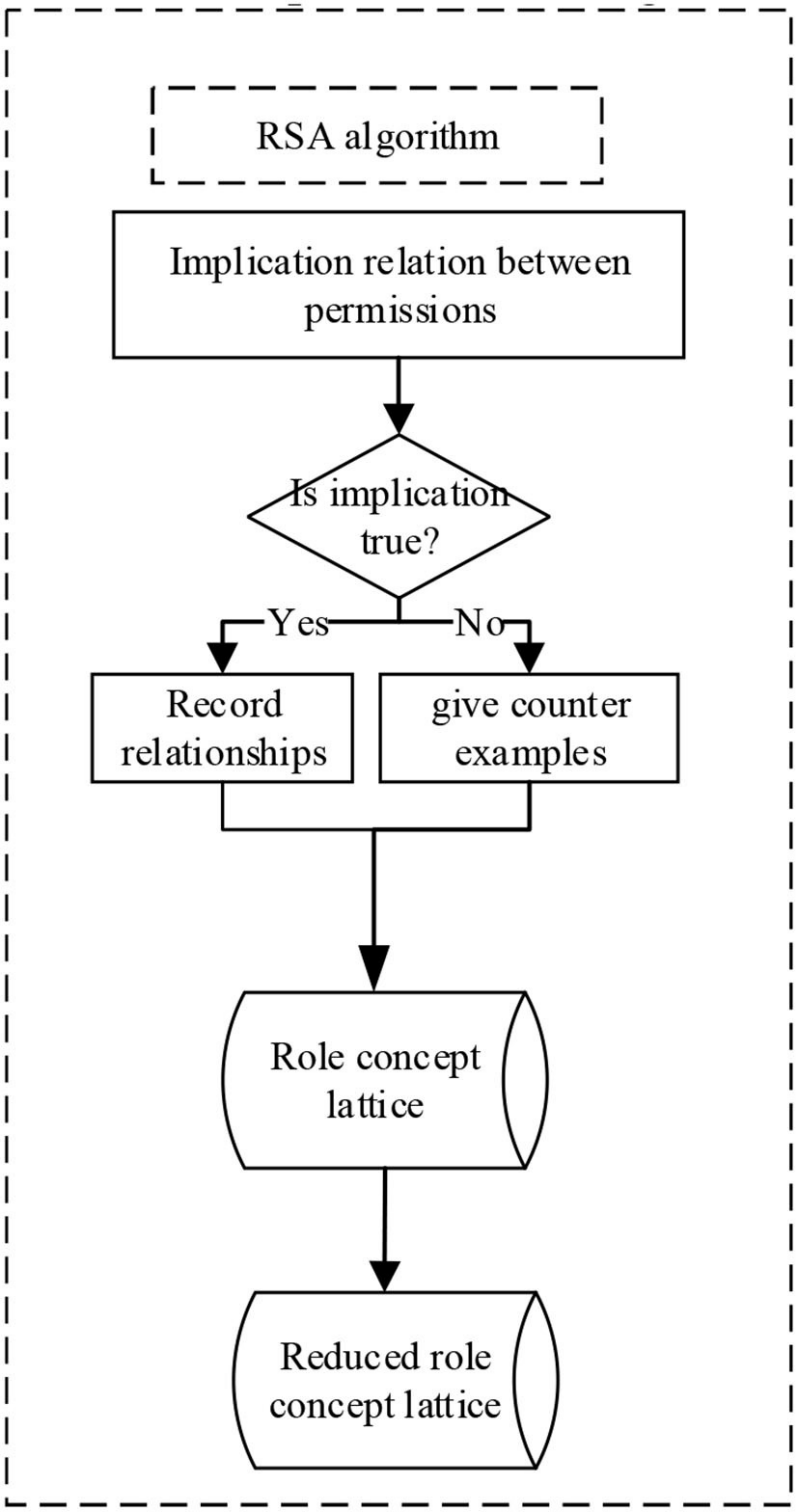
RSA algorithm.

The role concept lattice reduction algorithm, which is designed based on reducible attributes and reducible objects, further reduces the role concept lattice obtained by the role exploration method based on concept lattice, thus reducing the role system of the information system. The input of the algorithm is the concept lattice explored by the role exploration method based on the concept lattice, and the output is the reduced role concept lattice. The first and second lines of the algorithm divide concepts in concept lattice into intent and extension. The intent is composed of an attribute set, and the extension is composed of an object set. For the domain of access control, the intent is composed of a permission set, and the extension is composed of a user set. Lines 3–6 of the algorithm reduce several intents according to the definition of reducible attributes. Lines 7–10 of the algorithm reduce the collection of objects according to the reducible object definition. Finally, the reduced object and intent are recombined to obtain the reduced role concept lattice. The algorithm flow is shown in Figure 4.

## An example of the role optimization method for conceptual grain computing

This section uses an example to illustrate the running process of the RSA algorithm.

Let *K* = (*U, M, I*) be a formal context, where *U*=(1, 2, 3, 4, 5) and *M* = {*a, b, c, d, e*}. *Here, numbers* 1, 2, 3, 4, *and* 5 represent the dean of a college, dean of teaching, student, dean of scientific research, and dean of academic affairs, respectively. *Letters a, b, c, d, and e* represent enrollment information management, student registration information management, student status management, student curriculum review, and student curriculum development and modification, respectively. The relationship *I* between *U* and *M* is listed in Table 2 (*a* < *b* < *c* < *d* < *e*).

**Table 2:** Formal context **K**.

	**a**	**b**	**c**	**d**	**e**
1	1	1	0	1	1
2	1	1	1	0	0
3	0	0	0	1	0
4	1	1	1	0	0
5	1	1	0	0	0

The method of role concept lattice based on concept lattice is as follows:

The initial state is set as *K*_0_ = ∅, *C*_0_(*K*) = ∅, *J*_0_(*K*) = ∅, *B*_0_ = ∅ (refer to Table 3).

**Table 3:** Formal context **K**_**0**_.

	**a**	**b**	**c**	**d**	**e**

The counterexample object 1 that refutes the implication ø → abcde from K was removed and added to the formal background K_0 to get the formal background K_1 (refer to Table 4).

**Table 4:** Formal context **K**_**1**_.

	**a**	**b**	**c**	**d**	**e**
1	1	1	0	1	1

(1) In the formal context *K*_0_, *f*(*g*(∅)) = {*abcde*}. Judging ∅ → *f*(*g*(∅))−∅, i.e., ∅ → *abcde* holds true in *K*. For *g*(∅) = {1, 2, 3, 4} and *g*(*abcde*) = ∅ in K, considering {1, 2, 3, 4}⊈∅, ∅ → *abcde* does not hold in *K*. The counterexample object 1 that refutes the implication ∅ → *abcde* from K was removed and added to the formal context *K*_0_ to get the formal context *K*_1_ (refer to Table 4), where *C*_1_(*K*) = *C*_0_(*K*), *B*_1_ = *B*_0_, and *J*_1_(*K*) = *J*_0_(*K*).
(2) The process of the role exploration method is not described in this study.

**Figure 5:**
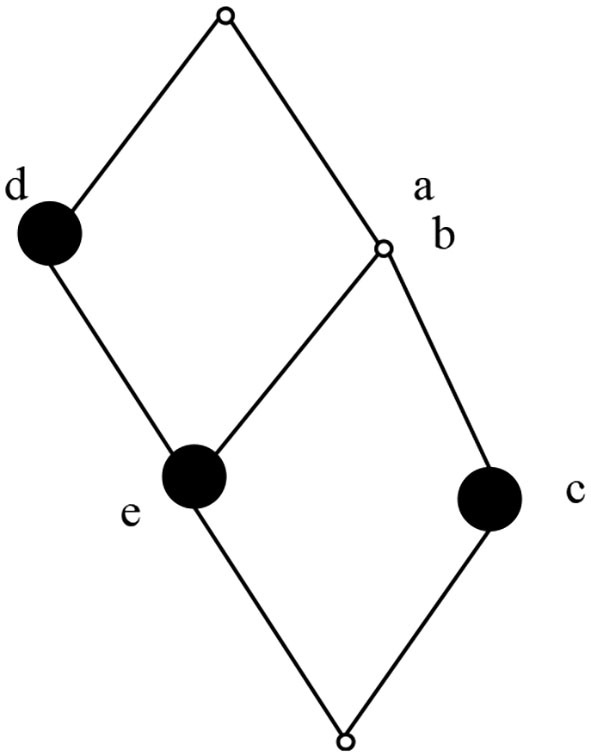
Role concept lattice.

The resulting intent set is {∅, *d, ab, abde, abc, abcde*}, and the concept set is {(1234, 5, ∅), (13, *d*), (124, 5, *ab*), (1, *abde*), (24, *abc*), (∅, *abcde*)}. The concept lattice composed of the above concepts is shown in Figure 5.

According to the principle of object reduction and attribute reduction, {*a, c, d*} and {*b, c, d*} are attribute particle reduction sets, where {*c, d*} are core attributes and {*a, b*} are relatively necessary attributes. {1, 2, 3} and {1, 3, 4} are object particle reduction sets, where {1, 3} are core objects and {2, 4} are relatively necessary objects. Therefore, object {5} and attributes are optimized and reduced to simplify the permissions and users on the role concept lattice, which is beneficial for system administrators to manage the system and reduce the complexity of the role system.

## Conclusion

At present, the role-based access control model occupies the mainstream position in the field of access control, but the role system for constructing RBAC is very complex. The role of an access control system can be obtained semiautomatically by using the concept lattice-based role exploration method, and the role concept can be used to obtain the role of an access control system, which is very simple and clear in form. Although the method can obtain the role system, when it is applied to the large-scale access control system, the scale of the role system obtained is also very large due to the large subject and object of access control, and the expansion of the role system makes the work of the security administrator increase dramatically. In this study, the theory of granularity computation and concept lattice reduction is introduced into the role exploration method based on concept lattice, and the role system obtained by the role exploration method is further optimized and reduced to obtain a smaller role system, which can also meet the security requirements of access control.

When the scale of the subject and object is very large, this method will be time-consuming. Therefore, the topology of concept lattice networks will be further considered in the following research (Liu et al., 2015, 2019) to better improve the time-consuming problem of this method. We will continue to explore and optimize the proposed method to further improve the robustness and performance of the algorithm. Considering that deep learning technology has been widely used in recent years (Zhang et al., 2020b, 2021a; Lv et al., 2021b, 2022, in Press), it will be introduced into access control in future research to speed up the processing speed. Therefore, the method proposed in this study can be parallelized in the next research, to better resolve the time-consuming problem of this method. In addition, it will be combined with privacy protection (Zhang et al., 2021b) to consider the practical application (Zhang et al., 2021) of the proposed method.

## Data availability statement

The original contributions presented in the study are included in the article/supplementary material, further inquiries can be directed to the corresponding author.

## Author contributions

All authors listed have made a substantial, direct, and intellectual contribution to the work and approved it for publication.

## Funding

This study was supported by the National Natural Science Foundation of China (Grant No. 42071318) and the National Defense Basic Research Projects of China (Grant No. JCKY2020908B001).

## Conflict of interest

The authors declare that the research was conducted in the absence of any commercial or financial relationships that could be construed as a potential conflict of interest.

## Publisher's note

All claims expressed in this article are solely those of the authors and do not necessarily represent those of their affiliated organizations, or those of the publisher, the editors and the reviewers. Any product that may be evaluated in this article, or claim that may be made by its manufacturer, is not guaranteed or endorsed by the publisher.
